# Management of locally advanced synchronous colorectal and prostate cancers: A case report

**DOI:** 10.1097/MD.0000000000020336

**Published:** 2020-05-29

**Authors:** Yi Qing Tey, Kavimalar Ravi, Choon Seng Chong, Edmund Chiong, Jingshan Ho, Jeremy Chee Seong Tey, Francis Ho

**Affiliations:** a Yong Loo Lin School of Medicine, National University of Singapore (NUS); b Department of Surgical Oncology (Colorectal Surgery); c Department of Surgical Oncology (Urology); d Department of Haematology-Oncology; e Department of Radiation Oncology, National University Cancer Institute, Singapore (NCIS), National University Health Systems (NUHS), Singapore.

**Keywords:** brachytherapy, cancer, colorectal, prostate, synchronous

## Abstract

**Introduction:**

Synchronous colorectal and prostate malignancies are uncommon, with standard treatment guidelines not yet established. Chemoradiation therapy is involved in both colorectal and prostate cancers. However, differing dosage regimens and effects of irradiation field on anatomical planes for surgery makes management of the synchronous cancers challenging. We report the first case of synchronous prostate and rectal cancer being treated with a combination of treatment modalities with a unique addition of high dose rate prostate brachytherapy boost.

**Patient concerns:**

The patient, a 69-year-old Chinese gentleman, presented with per-rectal bleeding with alternating bowel habits and a hemoglobin drop. He also had a history of urinary urge incontinence.

**Diagnosis:**

Following diagnostic workup, he was diagnosed with synchronous rectal adenocarcinoma (T3N1M0) and prostate malignancy (T2bN0M0).

**Interventions:**

The management consisted of neoadjuvant androgen deprivation therapy (ADT) and pelvic chemoradiation, followed by high dose rate prostate brachytherapy boost and subsequently anterior resection.

**Outcomes:**

Following therapy, the patient has no evidence of local recurrence or distant metastasis.

**Conclusion:**

We suggest a new feasible treatment strategy for the management of synchronous colorectal and prostate cancers.

## 1 Introduction

In Singapore, colorectal and prostate cancers are among the top three most frequently occurring in males.^[1]^ Although synchronous occurrences are not common, their concurrent incidence is expected to rise with improved screening and diagnostic modalities.^[2]^ There are well established separate guidelines for the management of colorectal and prostate cancers. However, care standards in treating synchronous malignancies have yet to be agreed upon.^[3]^ We report the first case of synchronous rectal and prostate carcinomas treated with neoadjuvant chemoradiation (NACRT) using external beam radiation therapy (EBRT), prostate brachytherapy boost with androgen deprivation therapy (ADT) and ultra-low anterior resection (ULAR).

## 2 Case report

### 2.1 Presenting concerns, clinical findings and diagnostic assessment

A 69-year-old Chinese gentleman with pre-existing comorbidities of type 2 diabetes mellitus, hyperlipidemia, and ischemic heart disease (on dual anti-platelet therapy status post-percutaneous coronary intervention) was diagnosed with T3N1M0 rectal adenocarcinoma and T2bN0M0 prostatic acinar adenocarcinoma (Gleason Score 3 + 4, intermediate risk group).

**Figure 1:**
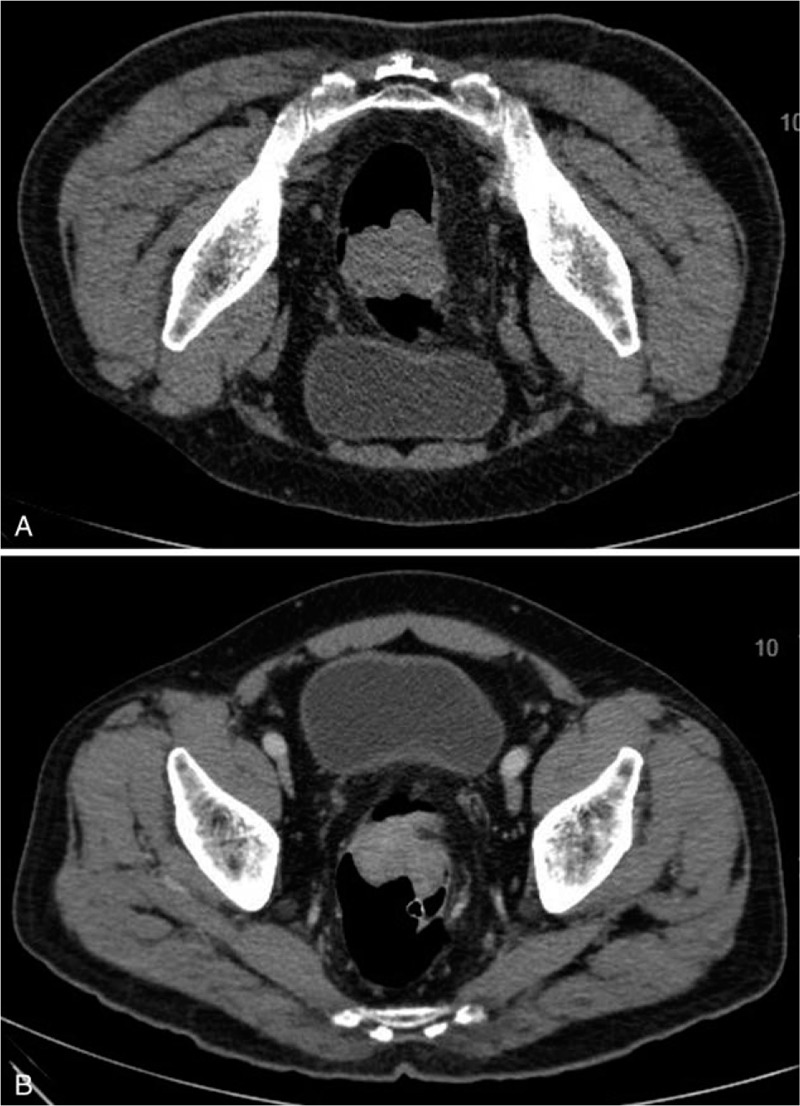
(A and B) CT colonography showing rectal adenocarcinoma.

**Figure 2:**
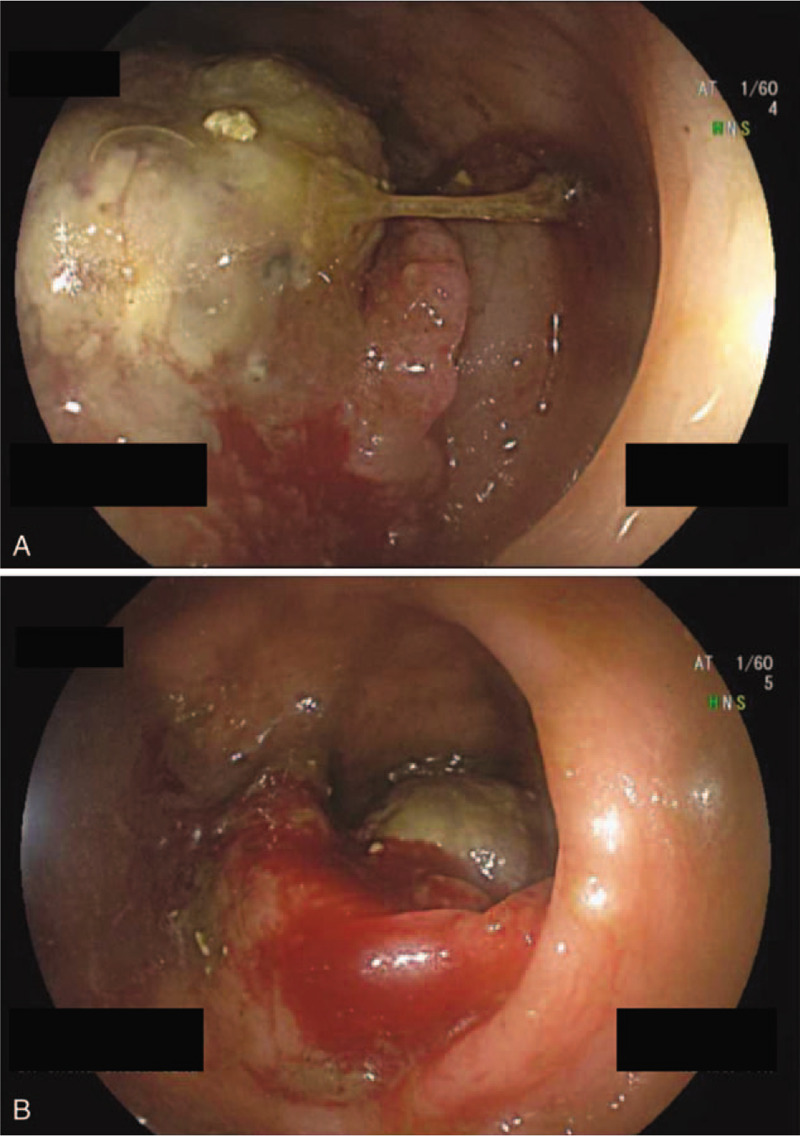
(A and B) Colonoscopy showing rectal adenocarcinoma.

He had initially presented with per-rectal bleeding which progressed to alternating diarrhoea and constipation with tenesmus. Digital rectal examination (DRE) yielded maroon stools and circumferential hemorrhoids with no active bleeding. There were palpable nodules on the right side of the prostate gland. The patient finally agreed to further investigations after a drop in hemoglobin (11.7–8.9 g/dL). Carcinoembryonic antigen (CEA) was not elevated at 3.8 ng/mL. Subsequent computed tomography (CT) colonography (Fig. 1A and B), colonoscopy with biopsy (Fig. 2A and B) and magnetic resonance imaging (MRI) of rectum confirmed his diagnosis of moderately differentiated rectal adenocarcinoma, with the lowest extent 7.5 cm from anal verge, with a craniocaudal extent of 3.8 cm. A few small volume mesorectal nodes were seen measuring up to 0.6 cm, as with several prominent superior rectal nodes measuring up to 0.6 cm. The rectal carcinoma was hence staged as cT3N1M0 according to American Joint Committee on Cancer (AJCC) TNM Staging 7th edition.^[4]^

**Figure 3:**
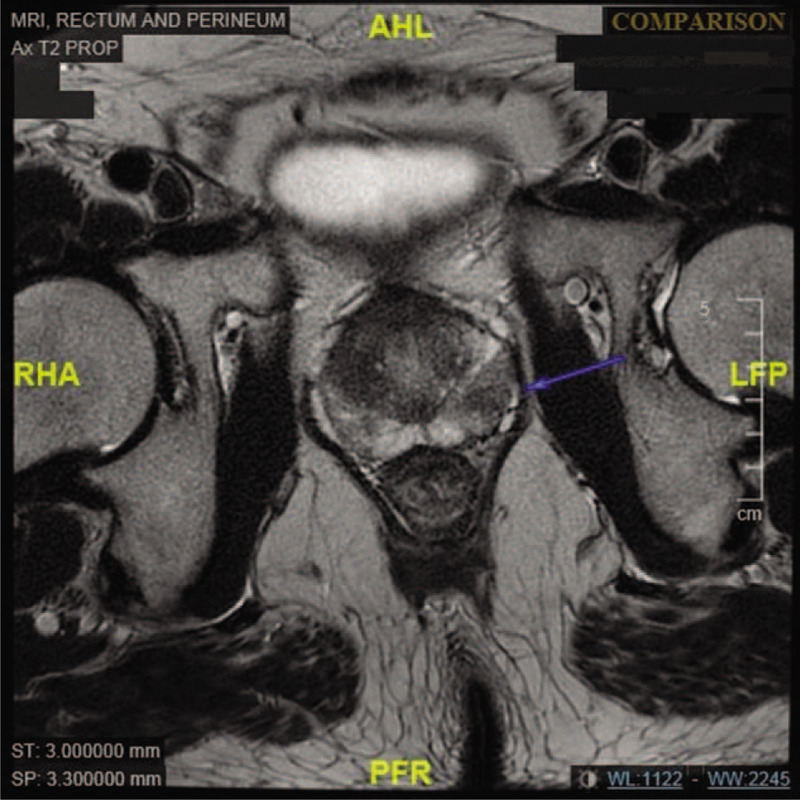
MRI showing prostate adenocarcinoma. Blue arrow indicating the prostate adenocarcinoma.

There was an incidental finding of mild prostatomegaly with underlying benign prostatic hyperplasia seen on MRI. Segmental T2w hypointensity was seen in the peripheral left prostatic lobe at the apex. The prostate was mildly enlarged measuring 5.6 × 3.4 × 3.1 cm, with a volume of 31 mL (Fig. 3). Urology workup ensued. Further history taking revealed a history of urinary urge incontinence. DRE revealed a nodule on the right prostate. Laboratory investigations revealed a raised prostate-specific antigen (PSA) of 20 μg/L. This prompted a trans-rectal ultrasound (TRUS) guided biopsy of the prostate gland, confirming the diagnosis of the intermediate risk prostatic carcinoma with a Gleason score of 3 + 4. There were 6 out of 6 cores positive for malignancy on the right while 1 out of 6 cores positive for malignancy on the left prostate gland. Based on the Memorial Sloan Kettering Cancer Centre (MSKCC) prostate cancer normogram, the risk of pelvic lymph node involvement was 10%, while the risk of extra capsular extension was 79%. The prostate carcinoma was, hence, staged as cT2bN0M0 according to AJCC TNM Staging 7th edition.^[4]^

Staging CT of the thorax, abdomen, and pelvis and a bone scan did not reveal any metastasis in the rest of the body.

### 2.2 Timeline

**Figure 4:**
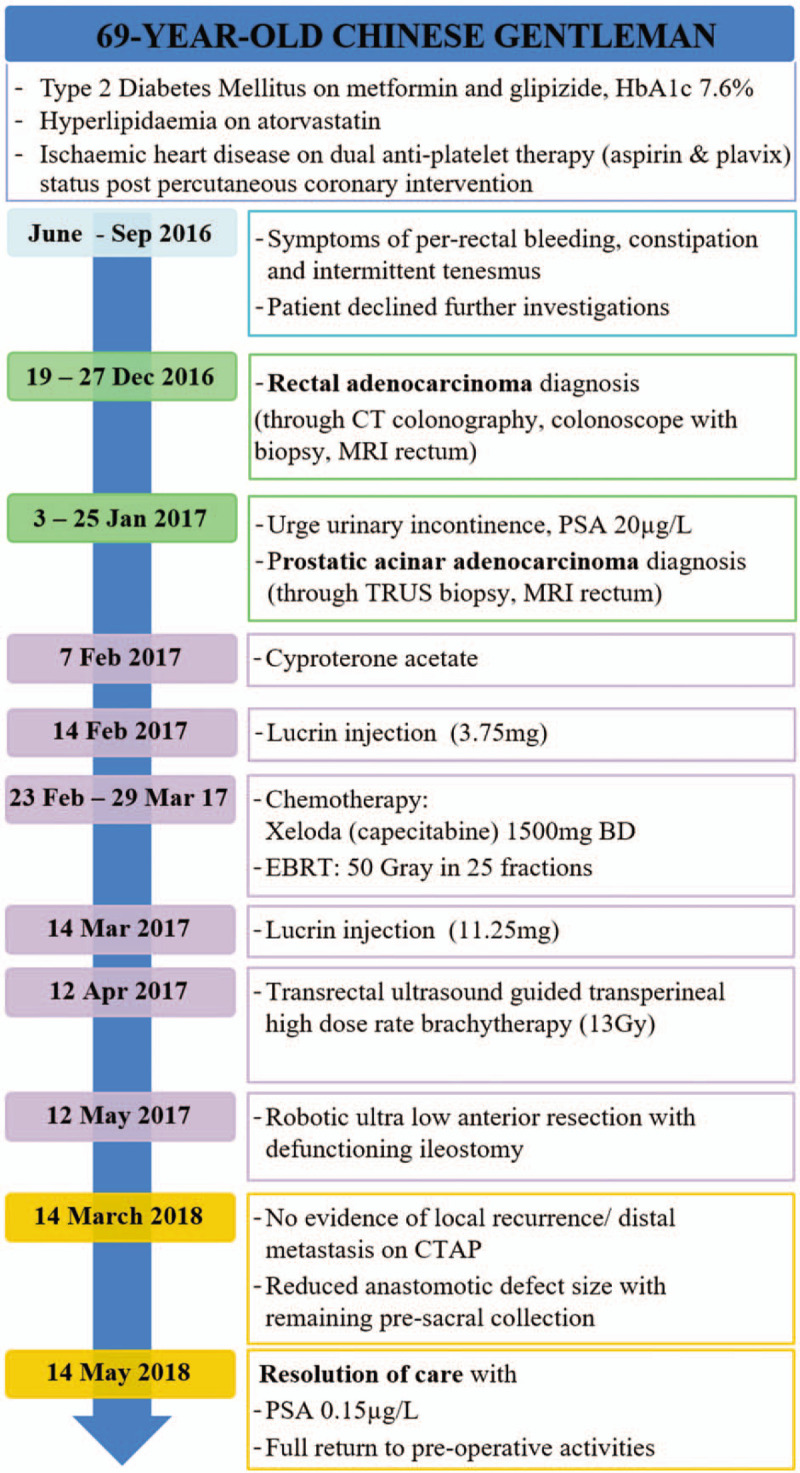
Timeline of patient's progress. Blue—symptoms; green—diagnoses; purple—treatment; orange—follow-up.

The patient's progress is shown in the timeline (Fig. 4).

### 2.3 Therapeutic intervention: multidisciplinary approach

He was seen by the urologist, colorectal surgeons, radiation oncologist, medical oncologist, and discussed at the urology and colorectal multidisciplinary tumor boards. The patient was given in the following options:

1. Robotic Ultra low anterior resection with defunctioning ileostomy and robot assisted radical prostatectomy (RARP) with pelvic lymph node dissection.
2. NACRT for rectal cancer followed by surgery (ULAR + RARP)
3. ADT and NACRT to pelvis (50 Gy) for rectal cancer and concurrent definitive radiation to prostate cancer (EBRT to 79.2 Gy) followed by ULAR.
4. ADT and NACRT to pelvis (50 Gy) for rectal cancer then interstitial high-dose rate prostate brachytherapy(13 Gy) followed by ULAR.

Pre-operative anesthetic review assessed him to be intermediate peri-operative cardiac risk for general anesthesia. The tumor board recommendation was for option 4. The options were discussed with the patient and he opted for option 4 after weighing the risks and benefits of each option.

Given the patient's premorbid status with good anal function, permanent colostomy was considered and offered but not preferred.

### 2.4 Treatment

The patient was treated with ADT and NACRT by EBRT (phase 1) with definitive prostate brachytherapy boost (phase 2) followed by an ULAR with defunctioning ileostomy (Table 1).

**Table 1:**
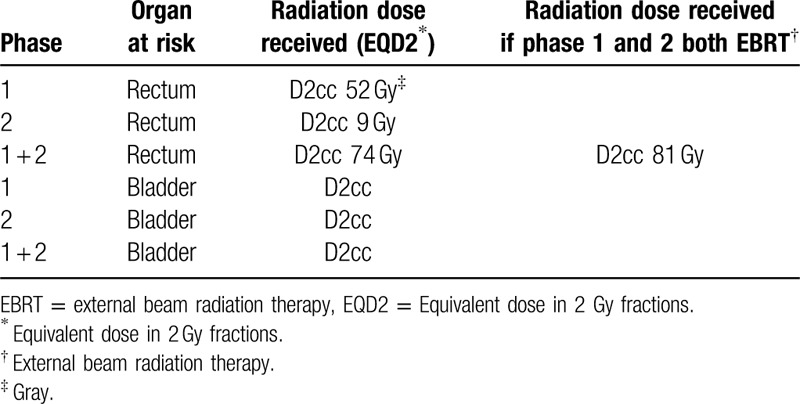
Organ at risk dose tolerances.

For his prostate carcinoma, he was started on Cyproterone acetate for 2 weeks followed by 2 Leuprorelin acetate depot injections 11.25 mg, which were administered 1 month apart for ADT.

In the management of the rectal adenocarcinoma, he underwent intensity modulated external beam radiotherapy with a total dose of 50 Gray (Gy) in 25 fractions over a period of 5 weeks. The clinical target included the entire prostate gland, seminal vesicles, rectal tumor and mesorectum, and pelvic lymph nodes. The pelvic lymph nodes were treated to a dose of 45 Gy in 25 fractions. This was delivered concurrently with Capecitabine of 1500 mg twice daily.

**Figure 5:**
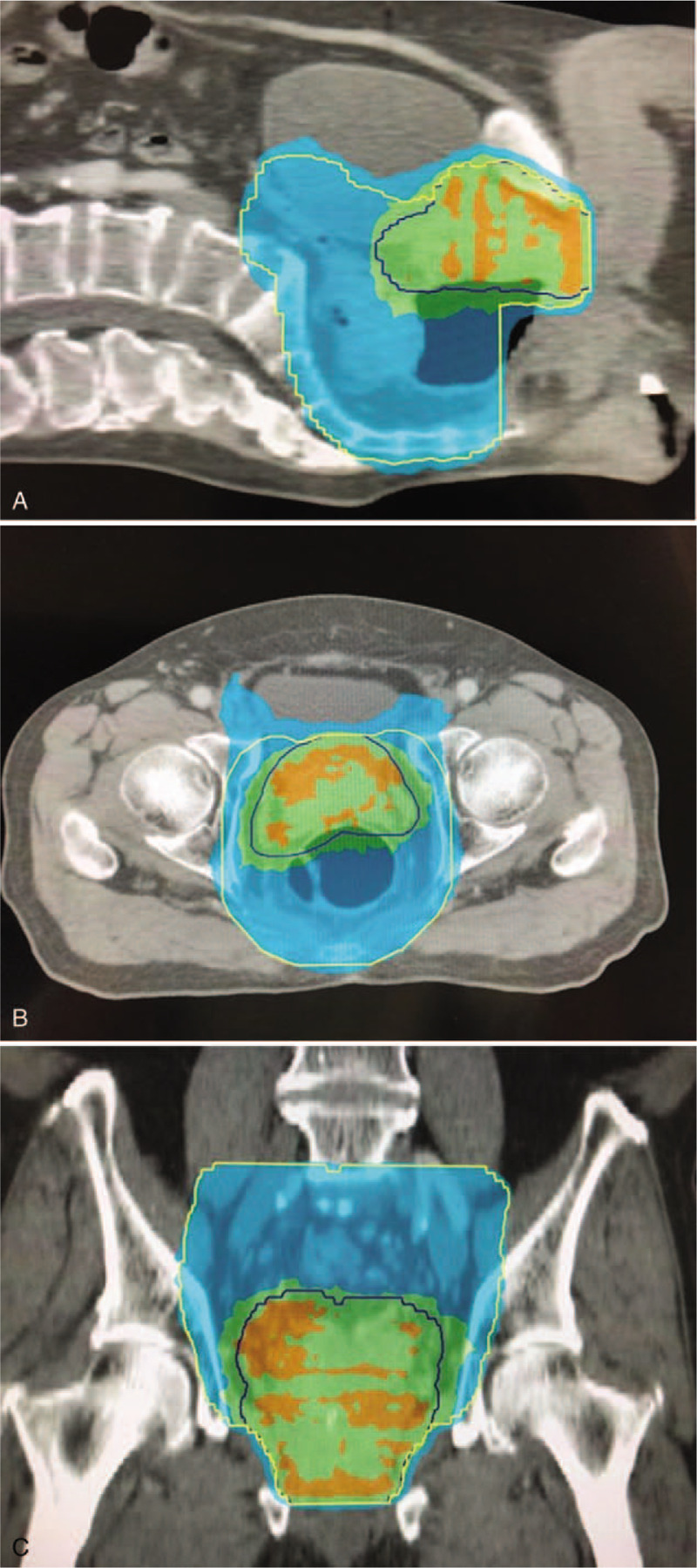
(A–C) Representative slices of radiotherapy plan. Yellow solid line—planning target volume for rectal tumor and pelvic lymph nodes; dark blue solid line—planning target volume for prostate carcinoma; blue color wash—45 Gy isodose; green color wash—50 Gy isodose; orange color wash—52 Gy isodose.

The representative slices of radiotherapy plan are shown in Figure 5A–C.

Following this, the prostate adenocarcinoma was treated with a transrectal ultrasound guided High Dose Rate brachytherapy using a transperineal approach. Severity of adverse events from the treatment was documented with the use of the Common Terminology Criteria for Adverse Events (CTCAE v4.0).^[5]^ The patient tolerated the therapy with grade 1 fatigue and grade 1 cystitis. One-month post-brachytherapy, the patient underwent ULAR with defunctioning ileostomy for the resection of the rectal carcinoma.

**Figure 6:**
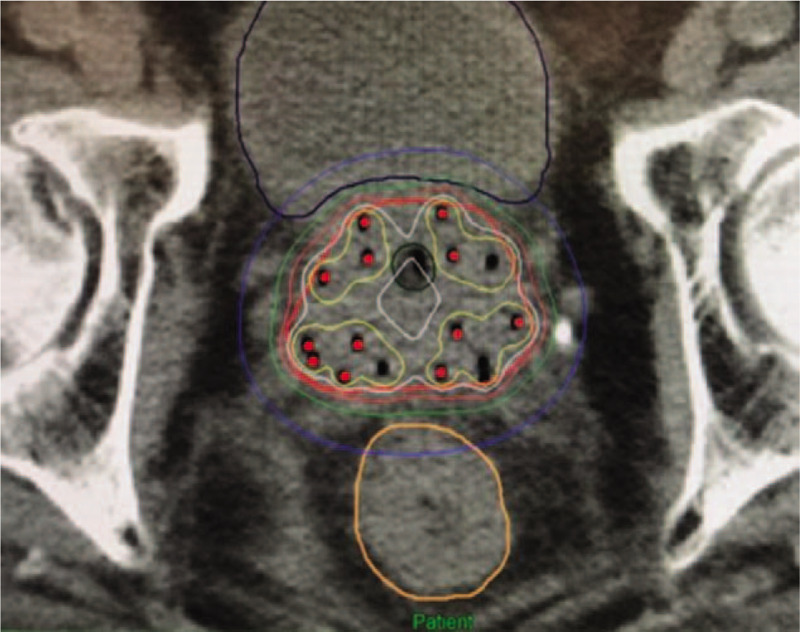
Representative slice of phase 2 brachytherapy plan. Red dots—brachytherapy source dwell locations; blue solid line—50% isodose line; green solid line—80% isodose line; red solid line—100% isodose line; liliac solid line—125% isodose line; yellow solid line—150% isodose line; orange solid line—rectum contour; dark blue solid line—bladder contour.

Representative slice of phase 2 brachytherapy plan is shown in Figure 6.

### 2.5 Treatment response

**Figure 7:**
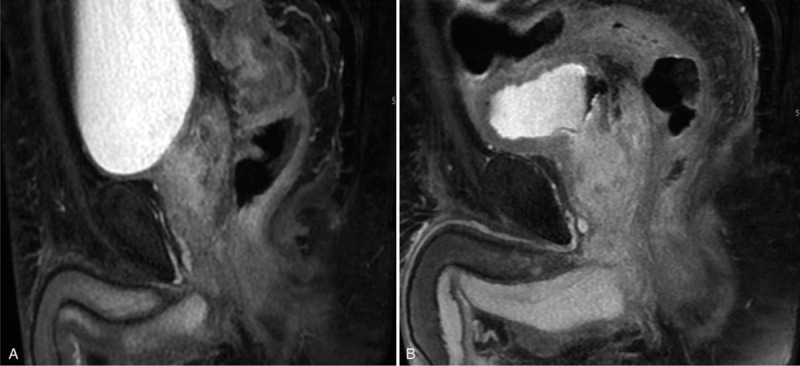
(A and B) MRI findings after neoadjuvant chemoradiation therapy.

A pathological complete response to neoadjuvant treatment was achieved for the rectal tumor with no residual tumor seen in the anterior resection specimen. This coincides with MRI findings after NACRT therapy (Fig. 7A and B). None of the 13 lymph nodes removed were involved by carcinoma. The pathological staging was hence ypT0N0M0 for the rectal cancer according to AJCC TNM Staging 7th edition.^[4]^

Post radiation PSA was 0.12 μg/L with a nadir of 0.11 μg/L at 8 months post-treatment.

### 2.6 Follow up and outcomes

**Figure 8:**
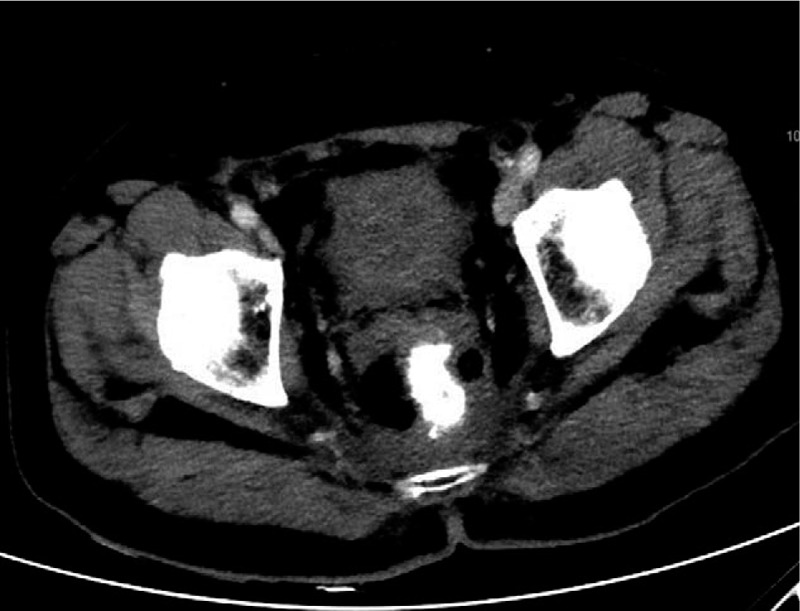
Surveillance CTAP done at 10 months post-operative follow-up.

Post-operatively, there was an anastomotic leak with pelvic collection. Adjuvant chemotherapy was not administered as a result. It was managed with transanal drainage and endoluminal vacuum therapy. There was no complication of pelvic sepsis. A residual 2 cm shallow anastomotic defect remains with no infection or mass. There is no evidence local recurrence or distant metastasis based on the surveillance CT Abdomen Pelvis (CTAP) done at 10 months post-operative follow-up (Fig. 8). At 1 year post-operation, the patient's PSA value remained low at 0.15 μg/L. He has now returned to his normal activities, with his weight returned to pre-morbid status and is satisfied with his outcome.

## 3 Discussion

Colorectal and prostate cancer are frequently occurring cancers in males worldwide. The standard of care in the treatment of locally advanced rectal cancer is NACRT followed by anterior resection which this patient received. There is no single standard of care in the treatment of intermediate risk prostate cancer. The following are all acceptable options and have been published prior.

- Radical prostatectomy (10–20% risk of requiring adjuvant radiotherapy) together with anterior resection and adjuvant treatment as needed.^[2,6]^
- NACRT to pelvis followed by external beam prostate boost radiation (using intensity-modulated radiation therapy [IMRT]), then anterior resection.^[3,7]^
- NACRT to pelvis followed by external beam prostate boost radiation (using volumetric modulated arc therapy [VMAT]), then anterior resection.^[8]^
- NACRT to pelvis followed by low dose rate prostate brachytherapy boost, then anterior resection.^[9]^
- NACRT to pelvis followed by high dose rate prostate brachytherapy boost, then anterior resection (our patient).

In general, the published literature suggests that the approach to synchronous rectal and prostate cancer has moved away from a surgery only approach.^[2,3,6–9]^ Prostatectomy after neoadjuvant radiation increases the technical difficulty of the prostatectomy^[3]^ and most urologists would avoid prostatectomy in a previous irradiated field unless no other treatment options are available.

Lin et al^[2]^ reported three patients who underwent radical prostatectomy with either an anterior resection or abdominoperineal resection in a single operation followed by adjuvant chemotherapy. The operative morbidity was reported to be low and out of the three patients, two remained asymptomatic and alive. Similarly, in Klee et al,^[6]^ three patients underwent a combined operation. One patient required adhesiolysis for small bowel obstruction and colostomy revision for ischemic colostomy. Another patient required dilatation due to development of strictures of the bladder and rectal anastomoses. All three patients remained cancer-free at 1 year follow-up. Formation of fistula between the bladder and the rectum is a potential risk in such combined operations. In our patient, however, his poor premorbid cardiac status made him an unsuitable candidate for a prolonged surgery like a combined prostatectomy and anterior resection. Consequently, the decision to treat the prostate cancer with definitive radiation was made.

Most centers now adopt an approach of NACRT consisting of pelvic RT followed by boost to the primary rectal tumor concurrent with oral capecitabine, followed by surgery in the form of anterior resection or abdominal perineal resection depending on the location of the rectal cancer.^[3,7–9]^ The reason for this is largely because of the proven benefit of neoadjuvant treatment in locally advanced rectal cancer.^[10]^ Moreover, there is also a role of pelvic irradiation in high risk prostate cancers.^[11]^

The main variation in this patient is in the way the prostate boost is delivered. As the availability of prostate brachytherapy is not as widespread as the accessibility to external beam radiotherapy, the larger series have all described a fractionated external beam boost dose to the prostate to a dose of between 20 and 28 Gy. Both intensity modulated radiotherapy and volume modulated arc radiotherapy have been utilized for the prostate boost with good outcomes.^[3,7,8]^ Lavan et al^[3]^ describes a retrospective review of ten patients who received pelvic EBRT with a dose of 45 to 50.4 Gy together with 5-fluorouracil with a total dose to the prostate of 70.0 to 79.2 Gy. No significant acute toxicities (grade 1 toxicity was the highest noted) were noted excluding grade 3 erectile dysfunction in patients who had received ADT. Nine out of these ten patients had surgery as one patient had declined surgery. Five patients were disease-free and four were alive with metastasis. Kavanagh et al^[7]^ identified five patients who were treated with a curative intent for their synchronous rectal and prostate cancers. Four of these patients received NACRT with pelvic EBRT (total dose of 74 Gy) with three experiencing grade 1 toxicities. This was followed by surgery for three patients and watchful waiting for one patient. The last patient underwent prostatectomy, NACRT and anterior resection. Three of these patients are alive with two who are disease-free. Ng et al^[8]^ describes the use of NACRT with EBRT using VMAT. The patient experienced low toxicity (grade 1) from treatment and subsequently underwent an abdominoperineal resection. Patient remains disease free at 1 year follow-up.

The use of prostate brachytherapy has been described in Qiu et al.^[9]^ In this case series, four patients were treated with NACRT to the pelvis, followed by a low dose rate brachytherapy boost followed by anterior resection and adjuvant chemotherapy. Three patients had grade 1 to 2 toxicities and one patient experienced grade 3 gastrointestinal toxicity. At 24- to 53-month post-treatment, one patient had rising CEA and one had metastatic prostate cancer. Prostate brachytherapy has the potential to spare the organs at risk and its larger radiation dose per fraction also overcomes the purported lower radiosensitivity of prostate cancer. This has led to a revolution in prostate cancer treatment in the last 2 decades. Despite the larger dose of radiation per fraction, the toxicity profile is largely considered superior in prostate cancer treatment. In a patient with prostate and rectal cancer treatment, the main toxicity is gastrointestinal toxicity in terms of stricture, bleeding and perforation. The key to reducing toxicity is therefore controlling the dose to the anastomosis as a large part of the rectum is removed in surgery. Hence, the colorectal surgeons’ involvement in the radiotherapy planning is important. The radiation field was planned with the intention of minimizing irradiation to the anastomotic area to minimize risk of anastomotic complications. The dose of brachytherapy was hence dropped from 15 to 13 Gy in order to meet the rectal dose constraints and to minimize risk of complications to the anastomosis.

However, despite our best efforts, the patient developed an anastomotic defect post-operatively. It was managed well with transanal drainage and Endoluminal Vacuum Therapy with no complication of pelvic sepsis. A few possibilities may account for this:

1. The patient's age is within the geriatric oncology age bracket, with significant comorbidities of diabetes mellitus.
2. Surgical factors could also have contributed.
3. Irradiation dose to the rectum was still suboptimal despite meeting internationally accepted radiation dose constraints.

Had we performed an external beam prostate boost, the dose to the rectum, would have been much higher, likely resulting in greater gastrointestinal toxicity. Perhaps a point of contention would be to drop the dose of the prostate brachytherapy boost further to further reduce toxicity. However, this would likely affect the outcome of his prostate cancer.

Using high dose rate brachytherapy as opposed to low dose rate brachytherapy also has a few advantages. From a radiation dosimetric perspective, it allows control over the post implant dosimetry through adjusting source dwell time, which is not possible in low dose rate once the seeds are in place. To the patient, high dose rate brachytherapy also avoids the inconvenience of having radiation sources in his body, hence has minimal disruption to the patient's lifestyle.^[12]^

## 4 Conclusion

We report the first case of synchronous prostate and rectal cancer being treated with the unique combination of neoadjuvant ADT and pelvic chemoradiation, followed by high dose rate prostate brachytherapy boost, then anterior resection is a feasible treatment strategy. Attention should be paid to the cumulative radiation dose to the site of eventual colorectal anastomosis to minimize gastrointestinal toxicity.

## Author contributions

**Conceptualization:** Choon Seng Chong, Edmund Chiong, Jingshan Ho, Jeremy Chee Seong Tey, Francis Ho.

**Investigation:** Choon Seng Chong, Edmund Chiong, Jingshan Ho, Jeremy Chee Seong Tey, Francis Ho.

**Methodology:** Choon Seng Chong, Edmund Chiong, Jingshan Ho, Jeremy Chee Seong Tey, Francis Ho.

**Writing – original draft:** Yi Qing Tey, Kavimalar Ravi, Francis Ho.

**Writing – review & editing:** Yi Qing Tey, Kavimalar Ravi, Choon Seng Chong, Edmund Chiong, Jingshan Ho, Jeremy Chee Seong Tey, Francis Ho.
